# Assessment of corneal vessels activity through the ‘Barcode sign’ of corneal OCT

**DOI:** 10.1038/s41433-024-03558-4

**Published:** 2025-01-25

**Authors:** Farida Omar Elzawahry, Prity Sahay, Dalia Said, Harminder Singh Dua

**Affiliations:** 1 Division of Clinical Neuroscience, Department of Ophthalmology, University of Nottingham, Nottingham, UK; 2 National Institute of Laser enhanced Sciences, Cairo University, Cairo, Egypt

**Keywords:** Corneal diseases, Eye manifestations

## Abstract

**Background/objectives:**

Anterior segment optical Coherence Tomography (AS-OCT) is used extensively in imaging the cornea in health and disease. Our objective was to analyse and monitor corneal vascularisation (CVas) through the corresponding back-shadows visible on AS-OCT.

**Subjects/Methods:**

AS-OCT scans were obtained from 26 consecutive patients (eyes) with CVas of different aetiologies. AS-OCT horizontal line scans showing the back shadows cast by the vessels were analysed and correlated with findings seen on slit lamp examination. Vessels were graded clinically as active, partially regressed, and regressed. The density of back shadow in the patient samples before and after treatment was analysed using Image-J software.

**Results:**

AS-OCT demonstrated a dense back shadow in all the 26 active vessels studied. When multiple vessels were present, the barcode sign was apparent. The back shadows absent in 22 (84.62%) at the regressed (healed) stage. The intensity of the backshadow had reduced in regressing vessels 4 (15.38%). The integrated density of the backshadow on AS-OCT in the active stage (pre-treatment) and corresponding healed (post-treatment, partially regressed or ghost vessels) scans was statistically significantly different (*P* < 0.0001).

**Conclusion:**

This study demonstrates that AS-OCT provides additional important information in patients with CVas enabling distinction between active and regressing/regressed vessels. Semiquantitative assessment can be made by measuring the integrated density of the back shadows produced by the vessels.

## Introduction

Anterior segment optical coherence tomography (ASOCT) has become a popular imaging tool for anterior segment imaging. Optical coherence tomography (OCT) is a non-contact modality of in-vivo ocular imaging. It uses low coherence interferometry that measures the echo time delay of light backscattered from tissue structures and combines multiple axial scans into a composite B-scan image, which delineates interfaces of differing refractive indices [1, 2]. Due to its quantitative and qualitative properties, ASOCT has been used to study various corneal pathologies such as keratitis, ectatic disorders, corneal dystrophies and degenerations, and ocular surface disorders [1, 3]. Recently, the ASOCT bar-code sign (BCS) of multiple corneal vessels was described [2]. A dark back shadow appears vertically across the corneal section posterior to the location of the vessel.

Corneal vascularization (CVas) is a sight-threatening condition affecting more than 1.4 million people per year. CVas results from a disruption in the balance of angiogenic and antiangiogenic factors that normally preserve corneal transparency [4, 5]. It occurs secondary to a wide variety of ocular insults, including hypoxia for contact lens overwear, infection, inflammation, ischaemia, degeneration, physical and chemical trauma, limbal stem cell deficiency (LSCD), pterygium and diffuse keratoconjunctival proliferation (DKP) [6–8]. Herpetic corneal disease is the commonest cause of CVas [9]. CVas, in turn, cause inflammation, lipid keratopathy, persistent oedema, and corneal haemorrhages and increase the risk of graft rejection. CVas are managed by different treatment modalities determined by the vessel activity [10]. AntiVEGFs are effective with active vessels whereas established (mature) vessels are best treated by fine needle diathermy occlusion [9]. Argon laser, collagen cross-linking, and intra-vascular mitomycin C have all been tried [4, 11–15].

Slit lamp bio-microscopy remains the instrument of choice to determine changes to the cornea, the technology has evolved over the years, and a detailed evaluation of anterior segment structures, with finer details than slit lamp bio-microscopy is achievable. Using indirect and retro-illumination can be used to detect lesions such as neovascularization, however as neovascularization can occur very rapidly, detection in early still poses a challenge [12, 16].

We undertook ASOCT examination of cases of established, resolving, and resolved CVas to establish features that can help in the monitoring and the evaluation of response to treatment, in these cases.

## Material and methods

The study was conducted at a university hospital clinical setting. Twenty-six eyes of 26 patients with corneal vascularisation of different aetiologies were studied.

ASOCT was carried out with a Heidelberg Spectralis (Spectralis HRA + OCT, Heidelberg, Germany) with the light beam at right angles to the major trunks of the corneal vessels giving cross-sectional images of the cornea. Twenty-one scans from limbus to the centre of the superior and inferior halves of the cornea were obtained for each eye. All Forty-two scans were examined individually for each patient and correlated with the slit lamp examination and images using (Topcon slit lamp SL-D701, Tokyo, Japan) as part of their routine assessment. Vessels were graded clinically as active (young and old), partially regressed, regressed and mature as previously described [10]. Briefly, corneal vessels were:

1. Active young vessels, which were freshly formed vessels that were full of blood, appeared bright red in colour, had minimal surrounding fibrous tissue sheathing and were actively progressing in the cornea with a well-defined arborising network of fine (capillary) vessels. The corneal stroma surrounding the vessels showed signs of leakage and oedema.
2. Active old vessels, which represented the stage when the vessels had reached and surrounded or covered the offending lesion in the cornea. Their progression ceased but consolidation continued. They appeared less bright than they originally did and maintained a brisk circulation.
3. Partially regressed, which was seen when the corneal pathology had abated in response to therapy or the arrival of corneal vessels or following fine needle diathermy of corneal vessels (FND). The circulation in the vascular complex was relatively slow, the vessels were less engorged and some parts of the complex had become less visible or underwent attrition. These vessels also showed arborisation.
4. Regressed (ghost vessels), these presented as fine white lines mirroring the morphology of the original vessels and were seen as ‘ghost vessels’. These did not have an active circulation and the cornea where they were located was not oedematous. All ghost vessels were located in the stroma.
5. Mature vessels, were relatively large vessels, with minimal arborisation and regressed or absent capillary networks, seen to persist in scar tissue or in the corneal stroma after the corneal pathology had healed. These vessels contained blood and maintained a circulation.

The density of back shadow in the patient samples (*n* = 26) before and after treatment were analysed using Image-J software (http://imagej.nih.gov/ij/; provided in the public domain by the National Institutes of Health [NIH], Bethesda, MD, USA) to obtain mean intensity, area, and integrated density. The mean of integrated density was plotted and expressed in arbitrary units (AU).

Statistical analysis was performed using GraphPad Prism 8.2.1 (GraphPad Software, Inc., San Diego, CA, USA). Descriptive statistics were computed for all continuous variables. The mean of the integrated density was analysed with column comparison and the data were represented as means ± standard deviation (SD). Data was analysed by the ANOVA paired *t*-test with a *p* value of < 0.05 was considered to be significant.

## Results

One thousand and ninety-two AS-OCT scans of twenty-six eyes corresponding to the active, and regressed stages of corneal vascularization were analysed. Twelve (44.4%) of them were females and 14 (53.8%) were males. The mean age of the patients was 45.85 ± 20 (25 years-91 years).

Six (23.07%) patients with limbal stem cell deficiency, four (15.38%) patients with diffuse keratoconjunctival proliferation (DKP), four (15.38%) patients with secondary vascularisation associated with graft rejection, 11 (42.30%) with corneal infection, including five (45.45%) herpetic, three (27.27%) fungal, three (gram-negative) bacterial (27.27%). (Table 1), and one (3.84%) patient with peripheral ulcerative keratitis (PUK), were retrospectively reviewed.

**Table 1:** Shows the underlying aetiology preceding corneal vascularization (CVas) and types of infective organisms.

Aetiology	Frequency
limbal stem cells deficiency (LSCD)	6 (23.07%)
Diffuse keratoconjunctival proliferation (DKP)	4 (15.38%)
Graft rejection	4 (15.38%)
Infection:	11 (42.30%)
Viral	5 (45.45%)
Bacterial (gram-negative)	3 (27.27%)
Fungal	3 (27.27%)
Peripheral ulcerative keratitis (PUK)	1 (3.84%)

### Active vessels

**Fig. 1:**
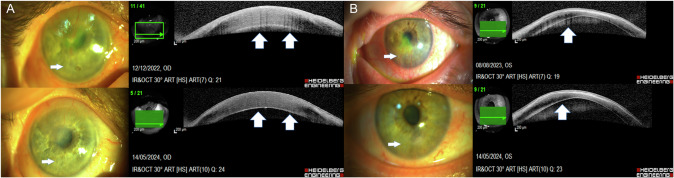
Slit lamp and OCT images of two cases of herpetic keratitis. **A** Slit lamp image of the right cornea showing a paracentral active herpetic infiltrate and active corneal vascularization (arrow, top left) and the slit lamp image showing regressed corneal vessels following treatment (arrow, bottom left). Corresponding anterior segment optical coherence tomograms (ASOCT) of the active vessels showing the barcode sign (BCS) produced by back-shadows of corneal vessels (block arrows, top right), and (ASOCT) of the regressed vessels showing a uniform iso-reflective cornea with no evident back-shadows in the same plane. (block arrows, bottom right). **B** Slit lamp image of the left cornea showing a paracentral active herpetic infiltrate and inferior active corneal vascularization (arrow, top left) and the slit lamp image showing a cleared cornea and regressed corneal vessels following treatment (arrow, bottom left). Corresponding anterior segment optical coherence tomograms (ASOCT) of the active vessels showing visible back-shadows of corneal vessels, despite the presence of a hypo-reflective tissue band cast by the overlying hyper-reflective infiltrate (block arrows, top right), and (ASOCT) of the regressed vessels not showing back-shadows over the hypo-reflective scar tissue shadow in the same plane. (block arrow, bottom right).

**Fig. 2:**
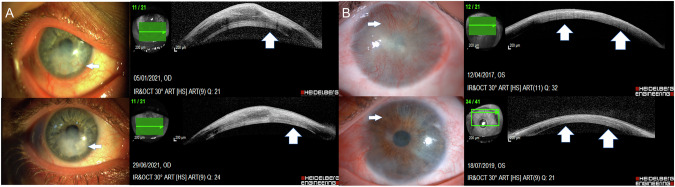
Slit lamp and OCT images of cases of fungal keratitis and diffuse keratoconjunctival proliferation (DKP). **A** Slit lamp and OCT images of a case of healed fungal keratitis. Slit lamp image of the right cornea showing scar tissue following a fungal keratitis ulcer, and blood vessels (arrow, top left), and a slit lamp image showing the same scar tissue area post fine needle diathermy (FND) and regressed vessels (arrow, bottom left). Corresponding anterior segment optical coherence tomograms (ASOCT) showing a thickened irregular cornea, with a hyper-reflective scar tissue band giving rise to multiple dark back-shadows corresponding to the active vessels (block arrow, top right), and ASOCT; central thinning and dense stromal hyper-reflectivity with no visible back shadow corresponding to regressed vessels post FND (block arrow, bottom right). **B** Slit lamp and OCT images of a case of diffuse keratoconjunctival proliferation (DKP). Slit lamp image of the right cornea showing scar tissue following a chemical burn with 360° limbal stem cell loss, and corneal conjuctivalization and vascularization. (arrow, top left), and a slit lamp image post-treatment showing a clear cornea and regressed vessels (arrow, bottom left). Corresponding anterior segment optical coherence tomograms (ASOCT) showing a superficial hyper-reflective scar tissue band giving rise to multiple dark back-shadows corresponding to the active vessels (block arrow, top right), and ASOCT; showing a thinned cornea and dense with no visible back shadow corresponding to regressed vessels (block arrows, bottom right).

The back shadow (barcode sign with multiple vessels) was noted in 26 (100%) eyes casting a dense back shadow at the active stage. The active vessels were bright red in colour with circulating blood and were clearly seen on both AS-OCT scans and slit lamp images (Figs. 1 and 2) (Supplementary Fig. 1).

**Fig. 3:**
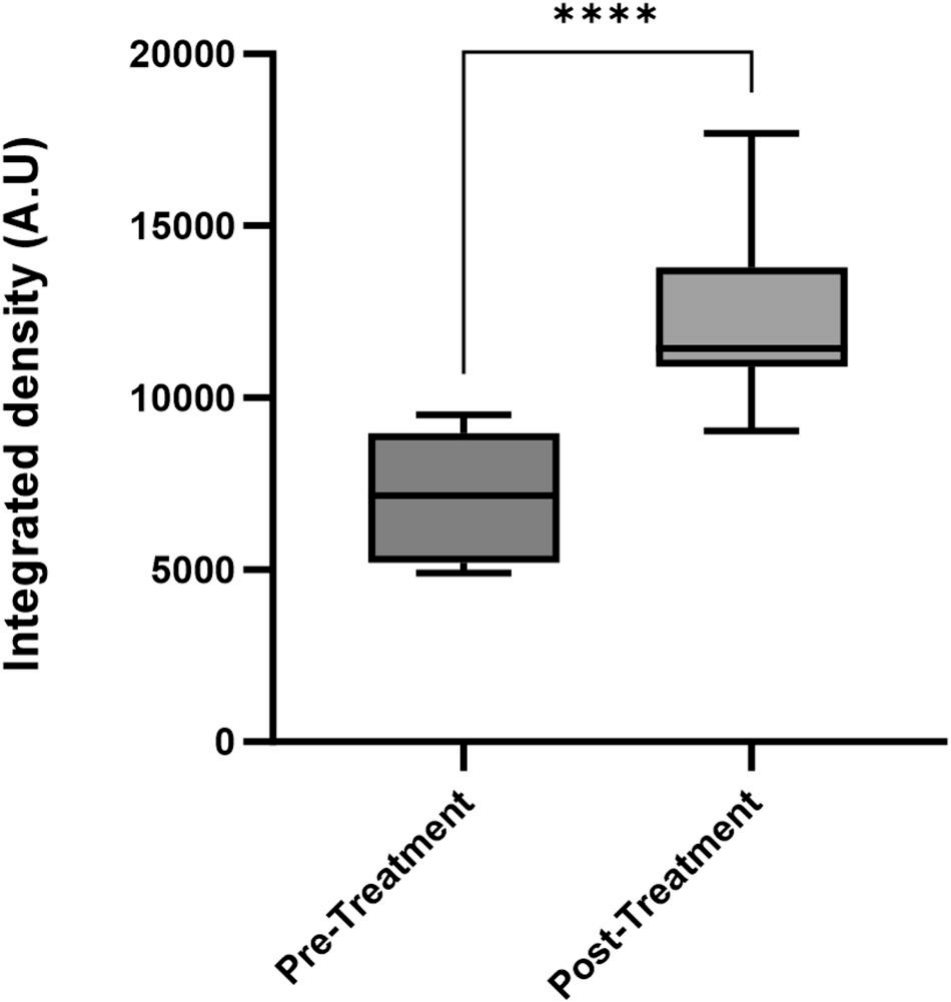
Data sets are plotted in box and violin graph indicating minimum and maximum values with standard deviation. Comparison of integrated density of the ASOCT vessels in the Pre-treatment, and Post-treatment groups showing a statistically significant *P* < 0.0001*.

### Regressed or partially regressed vessels

The back shadow (barcode sign) was absent in 22 (84.62%) at the regressed (healed) stage. Four (15.38%) at the regressing stage. These did not demonstrate any active circulation and showed less distinct back-shadow (Figs. 1, and 2).

The brightness or lack of it, in the area of back shadow on the ASOCT scan was measured as the Integrated density (IntDen) ± standard deviation. The IntDen of vessels on ASOCT in the active (pre-treatment, active stage) and corresponding healed (post-treatment, partially regressed or ghost vessels) scans were statistically significantly different (*P* < 0.0001) (Fig. 3).

## Discussion

CVas is a non-specific response to a variety of stimuli wherein abnormal blood vessels grow into the normally clear cornea. CVas is a double-edged sword, which on the one had is the body’s response to insult and helps in wound healing, combing invading organisms and eliminating noxious stimulants. On the other hand, CVas results in oedema, leakage of protein and lipids, perpetuate inflammation and affect the transparency of the cornea, affecting sight. Common causes of CVas include inflammatory conditions of the skin and connective tissues, infections, trauma, and certain ocular surface disorders [5–7, 16].

Various techniques are used in the evaluation and assessment of the extent, depth and severity of CVas. These include slit lamp biomicroscopy and photomicroscopy, videography, fluorescein angiography, OCT and optical coherence tomography angiography (OCTA), and corneal topography [12, 16, 17]. These modalities of assessment have several limitations such as imprecision, lack of quantification, difficulty of vessel delineation in areas of scarring, and evaluation of the blood flow within the vessels [18].

More recently, optical coherence tomography angiography (OCTA) has been established as a method for imaging retinal and corneal vessels. OCTA takes advantage of the high scan speed of Fourier-domain OCT for angiographic imaging and uses motion contrast to detect in vivo flow within blood vessels, without the need for contrast dye. This technology has been commercially available since 2014, however, much of its use has been limited to the research setting [19–21]. Furthermore, with the development of anti-angiogenic therapy, non-invasive techniques are needed for quantitative analysis in anterior segment vasculature. Binotti et al. and Ang et al. have used OCTA for the quantitative analysis of corneal neovascularisation to evaluate the extent of corneal NV and evaluate the severity of the condition [21–24]. However, anterior segment optical coherence tomography angiography (ASOCTA) evaluates a limited area and depth compared to ASOCT, which is designed for the external eye. To meet this challenge, ASOCT with angiography technology needs to be developed to help diagnosis and assessment of anterior segment diseases [24].

Unlike most studies on the use of ASOCT in corneal examination, our study was unique in that it monitored CVas through correlating ASOCT with the slit lamp examination and images. CVas presented as a hypo-reflective, dark back-shadow, which arises from depth of the location of major vascular trunks and extends throughout the entire corneal thickness. We have also established that the width of the shadow corresponded to the width of the vessel that casts the shadow. In multiple corneal NV, sequential vertical lines of back-shadows were clearly visible in a single scan, giving it the appearance of a bar code, previously described as the ‘bar code sign of corneal OCT’ [2].

Moreover, have been able to establish that the obstruction of the passage of light in ASOCT was mainly due to the circulating blood column (active vessels) rather than the vessel wall, as both arteries (afferent) and veins (efferent) have produced an equally dense back shadow despite their different vessel-wall structures. Our study also showed that partially regressed vessels as determined clinically by slit lamp imaging, cast a less dense back-shadow on ASOCT, and ghost vessels in cases of resolved CVas, which did not contain a circulating blood column, the back shadow was absent. Further evidence to demonstrate that the ‘regressed/closed’ vessels were not circulating blood comes from the observation that these vessels do not bleed during trephination for subsequent keratoplasty and that lipid keratopathy clears following fine needle diathermy occlusion of the vessels [14, 25].

We quantified this observation by measuring the integrated density in 26 ASOCT scans by measuring the density of the back-shadow cast by (active vessels) and (regressed vessels) [2]. Many studies have reported the attempt to clinically assess corneal NV in a qualitative or semi-quantitative manner based on the number of quadrants involved, depth of penetration and the centripetal progression. Vassileva et al., who determined the state of neovascularisation before and after intervention by comparing the size, number and centricity of vessels as well as the number of affected quadrants [26]. Similarly, Pillai et al. recorded the extent of corneal vascularisation with respect to the number of quadrants involved, the depth of vessels and whether they were active or quiescent [14].

Faraj et al. have also described the various clinical characteristics of corneal NV to standardise the clinical grading of corneal NV severity. Through their analysis of CVas images based on different parameters such as source, location and depth; number of quadrants affected; length (peripheral, mid-peripheral and central); branching pattern, leakage and nature of the blood flow. They also classified CVas into active young, active old, mature, partially regressed and regressed [10, 25].

In order to achieve a more accurate and quantitative measure of the extent of vascularisation, digital images provided the best way forward. In 1994, an in vivo automated method previously described the quantification of CVas based on contrast enhancement, followed by density threshold identification for the blood vessels and, finally, pixel measurement [27].

The same concept was adopted using ASOCT, by AlMaazmi et al. who studied aqueous leak in non-traumatic corneal perforations using ASOCT. The glue and corneal blood vessels consistently cast a dense shadow posteriorly. Relying on ASOCT they were able to differentiate between the back shadow cast by an active vessel and tissue glue through monitoring the density, width and depth of the shadow [28].

Varma et al. have used image processing software on ASOCT images in eyes with limbal stem cell deficiency (LSCD). They have described a method of quantifying the reflectivity patterns of the line scans of ASOCT and studies both epithelial and stromal reflectivities cast by scar tissue and/or corneal NV. However, as several corneal disorders with vascularization may mimic LSCD, they suggested that vessels in LSCD were mainly superficial and showed segmentation [29].

This study establishes a simple, semi-quantitative method using slit-lamp and ASOCT, to assess and monitor vessel activity. We have noted that all produced an equally dense back shadow, by comparing the integrated density in active (pre-treatment) and regressed (post-treatment) vessels. We inferred that the impediment to the passage of light was by the column of circulating blood in the vessels rather than the vessel wall. Hence, the presence of the ‘barcode sign’ was consistent with vessels with active circulation, and the clinical resolution, demonstrated by the reducing intensity or absence of the back shadow, corresponds to the low or absent blood flow in regressing and regressed vessels. This highlights the use of ASOCT in assessing CVas and their state of activity. In our personal experience, we have observed the back shadowing produced by corneal vessels with the Topcon OCT device (3D-OCT-2000) system (Topcon Corporation) and can also be seen in images in the published literature with the RTVue (Optovue, Inc., CA, USA) device [30].

Supplemental material is available at Eye’s website.

## Summary

### What was known before

- AS-OCT is used extensively in imaging the cornea. Defraction of light in the scans usually corresponds to differing in tissue density.
- AS-OCT highlights different corneal pathologies.

### What this study adds

- AS-OCT provides additional important information in patients with corneal vascularisation.
- AS-OCT enables distinction between active and regressing/regressed vessels.
- AS-OCT can Semiquantitatively assess the integrated density of the back shadows produced by the vessels.

## Supplementary information

Supplementary material discription (legends)

Supplementary figure 1

Supplementary video 1

## Data Availability

The datasets generated during and/or analysed during the current study are available from the corresponding author on reasonable request.

## References

[1] Gupta N, Varshney A, Ramappa M, Basu S, Romano V, Acharya M, et al. Role of AS-OCT in managing corneal disorders. Diagnostics. 2022;12:918. 35453966 10.3390/diagnostics12040918 PMC9030521

[2] El Zawahry FO, Beer F, Yildiz BK, Said DG, Dua HS. The ‘barcode sign’ seen on optical coherence tomography of extensive corneal vascularization. Eye. 2023;37:3863–4. 37524827 10.1038/s41433-023-02602-z PMC10698166

[3] Salim S. The role of anterior segment optical coherence tomography in glaucoma. J Ophthalmol. 2012;2012:476801. 22900146 10.1155/2012/476801 PMC3415232

[4] Sharif Z, Sharif W. Corneal neovascularization: updates on pathophysiology, investigations & management. Rom J Ophthalmol. 2019;63:15–22. 31198893 PMC6531773

[5] Beebe DC. Maintaining transparency: a review of the developmental physiology and pathophysiology of two avascular tissues. Semin Cell Dev Biol. 2008;19:125–33. 17920963 10.1016/j.semcdb.2007.08.014 PMC2276117

[6] Abdelfattah NS, Amgad M, Zayed AA, Salem H, Elkhanany AE, Hussein H, et al. Clinical correlates of common corneal neovascular diseases: a literature review. Int J Ophthalmol. 2015;8:182–93. 25709930 10.3980/j.issn.2222-3959.2015.01.32 PMC4325264

[7] Said DG, Mathew M, Shaikh MY, Maharajan VS, Lowe J, Dua HS. Diffuse keratoconjunctival proliferation: a novel clinical manifestation. Arch Ophthalmol. 2008;126:1226–32. 18779482 10.1001/archopht.126.9.1226

[8] Dua HS, Miri A, Said DG. Contemporary limbal stem cell transplantation - a review. Clin Exp Ophthalmol. 2010;38:104–17. 20398102 10.1111/j.1442-9071.2010.02229.x

[9] Schaub F, Hou Y, Zhang W, Bock F, Hos D, Cursiefen C. Corneal crosslinking ot regress pathologic corneal neovasvcularization before hing-risk keratoplasty. Cornea. 2021;40:147–55. 33395116 10.1097/ICO.0000000000002406

[10] Faraj LA, Said DG, Al-Aqaba M, Otri AM, Dua HS. Clinical evaluation and characterisation of corneal vascularisation. Br J Ophthalmol. 2016;100:315–22. 26163540 10.1136/bjophthalmol-2015-306686

[11] Mimouni M, Ouano D. Initial outcomes of mitomycin intravascular chemoembolization (MICE) for corneal neovascularization. Int Ophthalmol. 2022;42:2407–16. 35099664 10.1007/s10792-022-02240-6 PMC8801928

[12] Bachmann B, Taylor RS, Cursiefen C. Corneal neovascularization as a risk factor for graft failure and rejection after keratoplasty: an evidence-based meta-analysis. Ophthalmology. 2010;117:1300–5. 20605214 10.1016/j.ophtha.2010.01.039

[13] Gomer CJ, Ferrario A, Hayashi N, Rucker N, Szirth BC, Murphree AL. Molecular, cellular, and tissue responses following photodynamic therapy. Lasers Surg Med. 1988;8:450–63. 2976443 10.1002/lsm.1900080503

[14] Pillai CT, Dua HS, Hossain P. Fine needle diathermy occlusion of corneal vessels. Invest Ophthalmol Vis Sci. 2000;41:2148–53. 10892856

[15] Mousa HM, Saban DR, Perez VL. The cornea IV immunology, infection, neovascularization, and surgery chapter 1: corneal immunology. Exp Eye Res. 2021;205:108502. 33607075 10.1016/j.exer.2021.108502 PMC8462940

[16] Bock F, Onderka J, Hos D, Horn F, Martus P, Cursiefen C. Improved semiautomatic method for morphometry of angiogenesis and lymphangiogenesis in corneal flatmounts. Exp Eye Res. 2008;87:462–70. 18789928 10.1016/j.exer.2008.08.007

[17] Steger B, Romano V, Kaye SB. Corneal indocyanine green angiography to guide medical and surgical management of corneal neovascularization. Cornea. 2016;35:41–5. 26555579 10.1097/ICO.0000000000000683

[18] Ang M, Cai Y, Tan AC. Swept source optical coherence tomography angiography for contact lens-related corneal vascularization. J Ophthalmol. 2016;2016:9685297. 27752366 10.1155/2016/9685297 PMC5056277

[19] Greig EC, Duker JS, Waheed NK. A practical guide to optical coherence tomography angiography interpretation. Int J Retin Vitreous. 2020;6:55. 10.1186/s40942-020-00262-9 PMC7666474 33292740

[20] Tan ACS, Tan GS, Denniston AK, Keane PA, Ang M, Milea D, et al. An overview of the clinical applications of optical coherence tomography angiography. Eye. 2018;32:262–86. 28885606 10.1038/eye.2017.181 PMC5811700

[21] Binotti WW, Koseoglu ND, Nosé RM, Kenyon KR, Hamrah P. novel parameters to assess the severity of corneal neovascularization using anterior segment optical coherence tomography angiography. Am J Ophthalmol. 2021;222:206–17. 32822670 10.1016/j.ajo.2020.08.023 PMC11791782

[22] Ang M, Cai Y, Shahipasand S, Sim DA, Keane PA, Sng CC, et al. En face optical coherence tomography angiography for corneal neovascularisation. Br J Ophthalmol. 2016;100:616–21. 26311064 10.1136/bjophthalmol-2015-307338

[23] Luo M, Li Y, Zhuo Y. Advances and current clinical applications of anterior segment optical coherence tomography angiography. Front Med. 2021;8:721442. 10.3389/fmed.2021.721442 PMC8649770 34888319

[24] Venkateswaran N, Galor A, Wang J, Karp CL. Optical coherence tomography for ocular surface and corneal diseases: a review. Eye Vis. 2018;5:13. 10.1186/s40662-018-0107-0 PMC5996489 29942817

[25] Faraj LA, Said DG, Dua HS. Evaluation of corneal neovascularisation. Br J Ophthalmol. 2011;95:1343–4. 21937569 10.1136/bjophthalmol-2011-300856

[26] Vassileva PI, Hergeldzhieva TG. Avastin use in high risk corneal transplantation. Graefes Arch Clin Exp Ophthalmol. 2009;247:1701–6. 19680676 10.1007/s00417-009-1170-y

[27] Conrad TJ, Chandler DB, Corless JM, Klintworth GK. In vivo measurement of corneal angiogenesis with video data acquisition and computerized image analysis. Lab Investig. 1994;70:426–34. 7511717

[28] AlMaazmi A, Said DG, Messina M, AlSaadi A, Dua HS. Mechanism of fluid leak innon-traumatic corneal perforations: an anterior segment optical coherencetomography study. Br J Ophthalmol. 2020;104:1304–9. 31822464 10.1136/bjophthalmol-2019-315098

[29] Varma S, Shanbhag SS, Donthineni PR, Mishra DK, Singh V, Basu S. High-resolution optical coherence tomography angiography characteristics of limbal stem cell deficiency. Diagnostics. 2021;11:1130. 34205702 10.3390/diagnostics11061130 PMC8233779

[30] Sharma S, Murthy S. Anterior segment oct in diseases of the ocular surface and cornea. 2021. https://www.eophtha.com/posts/anterior-segment-optical-coherence-tomography-in-diseases-of-the-ocular-surface-and-cornea.

