# Supplementary material for: Assessment of corneal vessels activity through the ‘Barcode sign’ of corneal OCT
Source: Eye (Lond). 2025 Jan 25;39(7):1332–6. doi: 10.1038/s41433-024-03558-4 (PMC12043972; doi:10.1038/s41433-024-03558-4)
Supplement: Supplementary file 1 — Supplementary material discription (legends) [file 41433_2024_3558_MOESM1_ESM.docx]

Supplementary material:

Video: Left eye of a patient with an active kerato-conjunctival blood vessel encroaching on the lower nasal aspect of the cornea, showing a visible column of circulating blood.

Supplementary figure 1: Corresponding anterior segment optical coherence tomograms (ASOCT) with a line scan taken at the same level of the corneal aspect of the active blood vessel showing a dark back-shadow corresponding to the circulating blood column (arrow),
