# Supplementary figures and images for: Assessment of corneal vessels activity through the ‘Barcode sign’ of corneal OCT
Source: Eye (Lond). 2025 Jan 25;39(7):1332–6. doi: 10.1038/s41433-024-03558-4 (PMC12043972; doi:10.1038/s41433-024-03558-4)

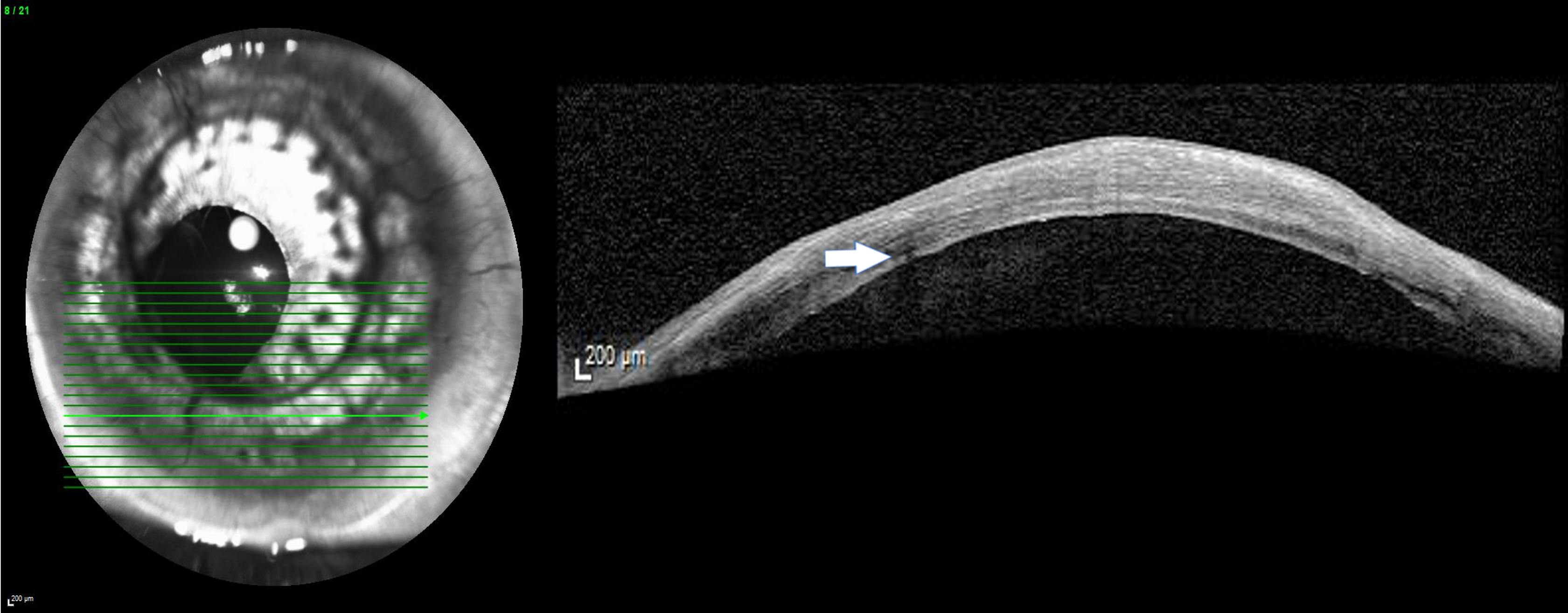

Supplement: Supplementary file 2 — Supplementary figure 1 [file 41433_2024_3558_MOESM2_ESM.tif]
